# The impact of sports experience on manual dexterity performances in school-age children

**DOI:** 10.1016/j.heliyon.2024.e41421

**Published:** 2024-12-21

**Authors:** Antonino Patti, Domenico Savio Salvatore Vicari, Valerio Giustino, Flavia Figlioli, Genny Margherita Seidita, Alessandra Calogera Montalto Monella, Aurora Oddo, Antonio Paoli, Patrik Drid, Ewan Thomas, Antonino Bianco

**Affiliations:** a Sport and Exercise Sciences Research Unit, Department of Psychology, Educational Science and Human Movement, University of Palermo, Palermo, Italy; b Department of Neurosciences, Biomedicine and Movement Sciences, University of Verona, Verona, Italy; c Department of Biomedical Sciences, University of Padova, Padova, Italy; d Faculty of Sport and Physical Education, University of Novi Sad, Novi Sad, Serbia

**Keywords:** Children, Fine motor skills, Manual dexterity, Fingers dexterity, Grooved pegboard test, Hand movement, Motor coordination, Physical activity

## Abstract

**Background:**

Manual dexterity is the ability to manipulate objects with precision and efficiency, using hands and fingers to achieve a specific objective. This study investigated how the practice of Capoeira, a sport that stimulates coordination skills, affects manual dexterity in children regularly engaged in physical activity or in sedentary children.

**Methods:**

Eighty-four participants were enrolled in this study, including forty-six males and thirty-eight females (age: 8.52 ± 1.52 years). They were divided into three groups: the Capoeira Group (n = 13), the Physical Activity Group (n = 30), and the Control Group (n = 41). Each participant completed a manual dexterity test (n = 5 trials) and the same test in dual-task (n = 2 trials) using the Grooved Pegboard Test (GPT).

**Results:**

The five trials (GPT1 to GPT5) showed a decrease in the time to complete the test by the participants. The between-group variance in the finger-tapping test (GPT-FTT) and the counting test (GPT-CT) showed significant differences between groups (<0.001).

**Conclusion:**

Our results indicate that children who practiced physical activity and Capoeira had higher levels of manual dexterity compared to those of the control group. This study indicates that structured sports, such as Capoeira, can have a beneficial impact on improving manual dexterity. Considering these findings, schools should support the development of fine motor coordination through physical activity programs that emphasize coordination tasks, such as Capoeira.

## 1 Introduction

Fine motor skills are essential for the healthy development of an individual. The literature suggests that using hands is fundamental for interacting with the environment. Fine motor skills are the ability to control and make precise hand movements to complete a task. They depend on the eyes and hands working together (visuomotor integration), the brain processing information about space and time (spatial-temporal integration), and the correct use of muscle strength for the task [[1], [2], [3]].

In 2021, Vaivre-Douret and colleagues, following previous studies, showed that graphomotor movements originate from phenotypic features in postural and functional kinetics [4]. They also noted that the shape of these movements can reflect developmental trajectories, thereby facilitating the assessment of graphomotor levels [4]. Furthermore, there is general agreement on the close correlation between advanced levels of gross and fine motor coordination in children and their academic success, improved cognitive abilities, and increased involvement in physical activities [2,3,5]. During school age, deficiencies in fine motor skills may impact daily activities and academic achievement [6]. Early fine motor skills in typically developing school-aged children have been linked to higher-order executive functioning abilities, including working memory and planning [7,8]. Early fine motor skills require the activation of basic and secondary motor brain networks and are significant indicators of academic preparedness. Overall, fine motor skills, such as manual dexterity, are closely related to neurological development and academic achievement [9]. Manual dexterity involves the ability to manipulate objects with precision and efficiency, using the hands and fingers for a specific purpose.

Some studies have analyzed the relationship between the practice of physical activity (PA), or participation in sports, and manual skills showing divergent results. Additionally, it remains unclear whether certain PAs are more effective than others in enhancing manual dexterity. Ziviani et al. (2009) examined the possible connection between daily PA and motor skills in children aged 6–12 years [10]. Their findings suggest that there is no substantial association between manual dexterity and daily PA. Similarly, in 2021, Kuloor observed no differences in hand-eye coordination between individuals who practiced sports and those who did not [11]. However, George et al. (2016) investigated the impact of a 6-week physical training, which incorporated multimedia tools, in youth between the ages of 6 and 12 years [12]. The researchers observed a notable improvement in manual dexterity performance, particularly in boys, but found no increase in the entire sample, which included both boys and girls [12].

Certain studies have investigated how sports can impact manual dexterity. Indeed, practicing a sport rather than another could improve manual dexterity. In 2023, Amato and colleagues suggested that basketball could promote the development of manual dexterity in young people [13].

Capoeira is an artistic expression of the Brazilian culture. It combines various components such as martial arts, dance, rhythm, and acrobatics [14,15]. Capoeira is characterized by its fluidity and improvisation, as participants engage in a rhythmic exchange of kicks, acrobatic movements, spins, and sweeps within a defined area, accompanied by music played on traditional instruments [16]. Finally, it qualifies as a sport due to its inclusion of specialized training in physical, technical, and tactical aspects [14]. Capoeira involves pairs of individuals participating more in a game than in a fight and it is known for its emphasis on agility and body coordination [[17], [18], [19]].

Given this information, it is essential to investigate manual dexterity during childhood and to understand the influence of PA and sport on the development and improvement of this skill. Various tools assess manual dexterity by measuring the speed and/or proficiency of hand and fingers movements. The Grooved Pegboard Test (GPT) is a commonly used test in the literature. The GPT requires the use of thumb and index finger to move grooved pegs and insert them into variously positioned holes on a pegboard [20].

This study aimed to examine and compare the impact of a specific sport, such as Capoeira, with PA and with a group of sedentary children on manual dexterity.

## 2 Materials and methods

### 2.1 Study design and procedure

**Fig. 1:**
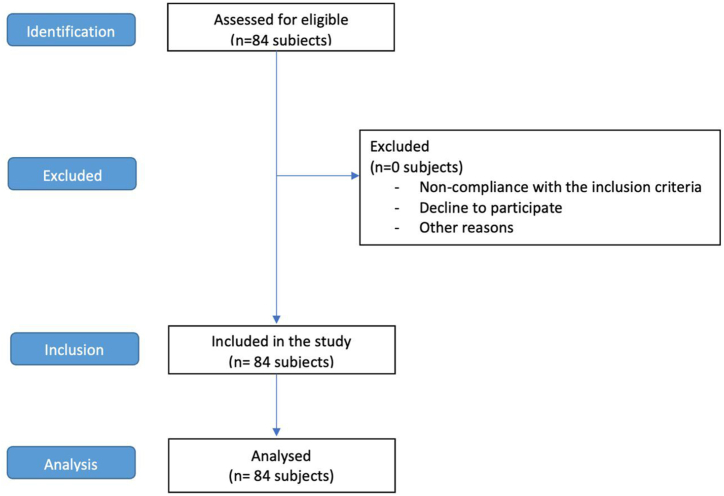
The STROBE flow chart.

The study design is an observational cross-sectional. The STROBE flow chart (Fig. 1) was used to ensure that the assessment of participants of the study was conducted clearly [21].

Following the school's participation, the study was presented to the children's parents, pointing out that participation would be voluntary, subject to the completion of an informed written consent.

The study was conducted following the recommendations of the Declaration of Helsinki for the involvement of people in research and was approved by the Bioethics Committee of the University of Palermo (Approval number: 88/2022).

### 2.2 Participants

Eighty-four participants were assessed for eligibility and enrolled in a school in Sicily (Italy). Forty-six boys and thirty-eight girls were recruited. The mean age of the sample was 8.52 years (±1.52). The mean age of the sample aligns with the age range reported in similar studies [[22], [23], [24], [25], [26]]. The participants were divided into three groups based on the type of PA or sports performed: the Capoeira Group (n = 13 participants); the Physical Activity Group (n = 30 participants) and, the Control Group (n = 41 participants).

Following the criteria outlined by Giustino et al. (2023), to be enrolled, participants had to meet the following exclusion criteria: a) upper limb injuries; b) mental disorders; c) neuromuscular/neurological diseases; d) drug therapy that could have affected neuromuscular or cognitive function [20].

### 2.3 Data collection

The data collection took place in a school classroom from 1:30 p.m. to 6:00 p.m. Participants were individually called from their respective courses and were tested one at a time, with each test session lasting about 15 min.

The protocol used was developed by Petrigna et al. (2020) and subsequently applied by Giustino et al. (2023) [20,27]. Placed on a desk, the GPT consisted of a tool consisting of pegs and holes arranged in random orientations within a 5 × 5 grid. Before starting, participants, seated in a chair near the desk, engaged in a familiarization session in which they filled only the first row of holes. They used their dominant hand to insert the pegs into the holes according to their orientation, proceeding from left to right and from top to bottom, completing the task as quickly as possible [20]. The protocol had two distinct phases: in the first, the participants completed five consecutive trials of the GPT (from GPT1 to GPT5) to assess the training effect. There was a 1-min interval between each trial. After a 3-min rest, participants performed a trial of the GPT combined with a motor task (i.e., finger tapping test; GPT-FTT) and a combined trial with a cognitive task (i.e., counting test; GPT-CT) to assess the participants' ability to perform both tasks simultaneously. There was a 1-min interval between trials. During both phases, the duration of each trial was recorded. The finger-tapping test consisted of constantly hitting the left hand's index on the desk. The counting test consisted of continuous counting from zero to ten and then back again. The order of the double-task trials (GPT-FTT and GPT-CT) was randomly assigned to the participants.

### 2.4 Statistical analysis

All data were recorded in an Excel file (Microsoft Corporation, Redmond, WA, USA). Descriptive statistics were carried out to present data as means and standard deviations. The Shapiro–Wilk normality test was used to analyze data distribution. The Levene's test was used to assess the homogeneity of variance of anthropometric characteristics between groups [28]. The repeated measures ANOVA with post-hoc was used to analyze the differences across the five consecutive trials and between groups. A post-hoc analysis was performed to calculate the power of the sample size recruited using the G∗Power software (version 3.1.9.2; Heinrich Heine University, Düsseldorf, Germany). Statistical analyses were performed using Jamovi software (version 2.3.21.0) with a p-value set significant at <0.05.

## 3 Results

The post-hoc analysis of the power of the sample size recruited showed a power = 0.98 (f = 0.25; α = 0.05). The Shapiro-Wilk test showed that the data were not normally distributed for all variables.

For this study, we enrolled 84 participants (age (y): 8.52 ± 1.52; height (cm): 132 ± 13.8; weight (kg): 32.5 ± 11.7). The Levene's test computed for anthropometric characteristics of the participants indicated that the variances were equal (p > 0.05) confirming the condition for homogeneity of the sample. The description of the participants' characteristics of each group is presented in Table 1.

**Table 1:** Anthropometric characteristics of the participants and homogeneity between groups.

	Physical Activity Group (n = 30)	Capoeira Group (n = 13)	Control Group (n = 41)	Levene's test (p)
Age (years)	8.40 ± 1.40	9.69 ± 1.70	8.24 ± 1.41	0.410
Height (cm)	136 ± 13.1	143 ± 15.4	126 ± 10.2	0.194
Weight (kg)	35.5 ± 12.4	38.7 ± 11.9	28.3 ± 9.50	0.099

Table 2 shows the performances of the sample of the five consecutive trials (from GPT1 to GPT5). These performances exhibit a decline across the five trials.

**Table 2:** Performances of the five consecutive GPT trials of the entire sample.

	GPT1 (s)	GPT2 (s)	GPT3 (s)	GPT4 (s)	GPT5 (s)
Participants (n)	84	84	84	84	84
Mean	90.9	79.7	77.9	75.3	74.0
Standard deviation	22.4	16.9	18.4	16.1	14.1

The impact of PA and Capoeira was also found to be significant. Nevertheless, the effect, as shown by η^2^, is relatively minimal. Table 3 provides a comprehensive analysis of variance.

**Table 3:** Repeated measures ANOVA between the performances of GPT1 to GPT5 with and without the influence of the sport and physical activity.

**Repeated Measures ANOVA**						
**Between Subjects Effects**	**Sum of Squares**	**df**	**Mean Square**	**F**	**p**	**η**^**2**^
Activity	44074	2	22037	25.8	<0.001	0.314

**Within Subjects Effects**	**Sum of Squares**	**df**	**Mean Square**	**F**	**p**	**η**^**2**^
GPT 1-5	9225	4	2306.3	45.55	<0.001	0.066
GPT 1–5 ✻ Activity	1440	8	180.0	3.56	<0.001	0.010

The variance between groups in the GPT-FTT and the GPT-CT performances is presented in Table 4, Table 5.

**Table 4:** Repeated measures ANOVA between the GPT-FTT performances of the groups.

ANOVA - GPT-FTT (s)						
Activity	**Sum of Squares**	**df**	**Mean Square**	**F**	**p**	
	5052	2	2526	10.4	<0.001	
POST HOC TESTS						

Physical Activity Group vs Capoeira Group	**Mean difference**	**SE**	**Df**	**T**	**B**_**onferroni**_	**Cohen's d**
	12.60	5.16	81.0	2.44	0.051	0.810
Physical Activity Group vs Control Group	−9.31	3.74	81.0	−2.49	0.044	−0.599
Capoeira Group vs Control Group	−21.91	4.95	81.0	−4.43	<0.001	−1.409

**Table 5:** Repeated measures ANOVA between the GPT-CT performances of the groups.

ANOVA - GPT-CT (s)						
Activity	**Sum of Squares**	**df**	**Mean Square**	**F**	**p**	
	7340	2	3670	19.4	<0.001	
POST HOC TESTS						

Physical Activity Group vs Capoeira Group	**Mean difference**	**SE**	**Df**	**T**	**B**_**onferroni**_	**Cohen's d**
	11.8	4.56	81.0	2.59	0.034	0.861
Physical Activity Group vs Control Group	−13.4	3.30	81.0	−4.07	<0.001	−0.977
Capoeira Group vs Control Group	−25.3	4.37	81.0	−5.78	<0.001	−1.839

**Fig. 2:**
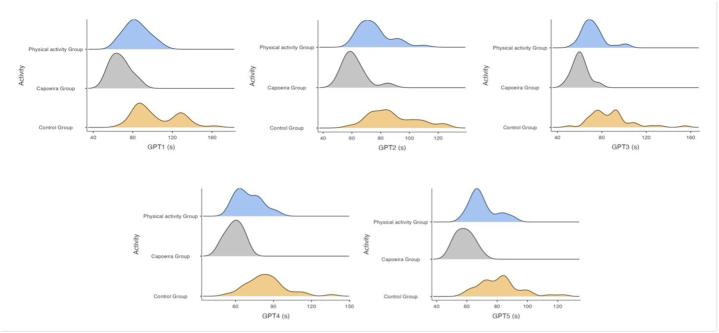
Density plots showing the distribution of the five sequential GPT performances of the three groups.

The statistical analysis revealed that the Capoeira Group had higher performances compared to both the Physical Activity Group and the Control Group in both tests. Our data indicate that PA also has a beneficial impact on manual dexterity but with a lower magnitude. Furthermore, the density plots in Fig. 2 describe that the performance measured in the five consecutive GPT trials are more uniform in the Capoeira Group.

## 4 Discussion

The study aimed to investigate and compare the impact of PA and a specific sport, such as Capoeira, on manual dexterity. Our findings demonstrated a significant improvement in the performance of the five consecutive GPT trials. A notable improvement in the performance across the five trials may suggest a learning of skills and its improvement [[29], [30], [31]]. Although the repeated measures ANOVA was statistically significant, the effect size was relatively small, as reported in Table 3. Nevertheless, it is essential to consider that physical stress could impact the GPT performance, and our results could potentially be underestimated due to the aforementioned stress [32]. The literature suggests that children have a heightened sensitivity to stress resulting from performance demands and adult expectations. This sensitivity may arise from several sources, such as the desire to satisfy authority figures, fear of failure, and evolving feelings of self-esteem and self-efficacy [33].

We evaluated the participants’ manual dexterity performance simultaneously with the execution of a motor task (GPT-FTT) and a cognitive task (GPT-CT) with the aim of determining whether the practice of PA or sport could influence their ability to handle objects in dual-task [27,34,35]. The results of the GPT performances in dual-task (both in GPT-FTT and GPT-CT) showed that participants who practiced PA or Capoeira had higher performances than the control group. Furthermore, children who practiced Capoeira performed considerably better than those involved in PA. To enhance the development of gross motor skills in school-aged children, it is crucial to implement their PA level by engaging them in activity for a minimum of 60 min each day, ideally at a moderate-to-vigorous intensity [36]. However, when performing an in-depth analysis, it is clear that some elements of global motor skills may not be sufficiently stimulated, or stimulated equally, during the practice of general PA since it cannot be exclusively assessed through quantity and quality [37]. In a study conducted by Biino et al. (2023), it was shown that at the age of 8–10 years old, structured sports activities have already a specific impact on the development of motor coordination based on the sport practiced [22].

This study has the following limitations. The recreational and amateur nature of the PA and the age of the participants made it difficult to quantify years of practice or continuity of PA practice. Despite the sample size power reaching 98 %, we argue that the Capoeira group had a relatively limited number of participants. In addition, although the results of the GPT performance test for the total sample across the five consecutive trials (GPT1 to GPT5) showed a significant difference between groups, and the density graphs revealed that the performances of the Capoeira group were more uniform than those of the other groups, the impact of sports practice did not detect a satisfactory effect size. Further studies involving larger cohorts will be essential to delve deeper into these aspects. Moreover, further studies should examine the performance model of Capoeira, a sport that is poorly explored in the scientific literature.

Our findings suggest that a structured sport which emphasizes motor coordination, such as Capoeira, have a beneficial effect on manual dexterity in school-aged children. It is crucial that the school environment is dedicated to promoting the growth of these skills to support their optimal physical development.

## CRediT authorship contribution statement

**Antonino Patti:** Writing – original draft, Methodology, Formal analysis, Conceptualization. **Domenico Savio Salvatore Vicari:** Writing – review & editing. **Valerio Giustino:** Writing – review & editing, Methodology, Conceptualization. **Flavia Figlioli:** Data curation. **Genny Margherita Seidita:** Data curation. **Alessandra Calogera Montalto Monella:** Data curation. **Aurora Oddo:** Data curation. **Antonio Paoli:** Visualization, Validation. **Patrik Drid:** Visualization, Validation. **Ewan Thomas:** Formal analysis. **Antonino Bianco:** Supervision, Conceptualization.

## Data availability statement

The datasets generated during the current study are available from the corresponding author upon reasonable request.

## Funding

This study did not receive any funding.

## Declaration of competing interest

The authors declare that they have no known competing financial interests or personal relationships that could have appeared to influence the work reported in this paper.
